# Awareness, attitudes and early use of doxycycline prophylaxis among gbMSM in Ireland: findings from a 2025 community-based cross-sectional survey

**DOI:** 10.1136/sextrans-2025-056745

**Published:** 2026-01-14

**Authors:** John Gilmore, David J Field, Helen Kelly, Robert Lawlor, Chris Noone, Michael Traeger, John White

**Affiliations:** 1 University College Dublin, Dublin, Ireland; 2 Gay Men’s Health Service, HSE, Dublin, Ireland; 3 UCD School of Public Health Physiotherapy and Sports Science, Dublin, Ireland; 4 University of Galway School of Psychology, Galway, Ireland; 5 Burnet Institute, Melbourne, Victoria, Australia; 6 HSE North West, Galway, Ireland; 7 Northern and Western Health and Social Care Trust, Health and Social Care Northern Ireland, Derry, UK

**Keywords:** antibiotic prophylaxis, sexual health, homosexuality, male

## Abstract

**Abstract:**

**Background:**

Bacterial sexually transmitted infections (STIs) remain a growing public health challenge globally, with gay, bisexual and other men who have sex with men (gbMSM) disproportionately affected. Doxycycline postexposure prophylaxis (DoxyPEP) has been shown in clinical trials to reduce syphilis and chlamydia, and has been incorporated into US and UK guidelines. However, community-level data in many European countries remain scarce. This study aimed to assess awareness, attitudes and early use of DoxyPEP among gbMSM in Ireland.

**Methods:**

An anonymous, cross-sectional online survey was conducted between May and June 2025. Eligible participants were aged ≥18 years, identified as male (cis or trans) or non-binary/gender diverse and reported sex with a man in the past 12 months. The questionnaire covered demographics, sexual behaviours, STI/HIV history, awareness and use of antibiotics for STI prevention and attitudinal measures. Descriptive statistics summarised findings, and logistic regression identified predictors of DoxyPEP use.

**Results:**

A total of 149 participants completed the survey, with a mean age of 36.4 years (range: 22–67); 92.6% were cisgender men and 86.6% identified as gay. Awareness of antibiotic STI prophylaxis was high (83.2%), and 69.1% expressed strong interest in future use. Over one-quarter (29.5%) reported DoxyPEP use in the past 12 months, almost exclusively at the recommended 200 mg dose. DoxyPEP use was associated with previous HIV PEP use (adjusted OR (AOR) 3.02 s, 95% CI 1.35 to 6.73) and group sex (AOR 3.27, 95% CI 1.26 to 8.59). Most participants reported sourcing antibiotics informally, including online or through friends. Antimicrobial resistance was the most common concern reported (69.8%).

**Conclusion:**

Despite the absence of national guidelines on the use of DoxyPEP for STI prevention, over one-quarter of participants reported using DoxyPEP, with high awareness and demand for structured access. These findings highlight the urgency for evidence-based, internationally aligned policies that ensure safe and equitable delivery, integrated within sexual health services and underpinned by antimicrobial stewardship.

**WHAT IS ALREADY KNOWN ON THIS TOPIC**

- Doxycycline post-exposure prophylaxis (DoxyPEP) reduces syphilis and chlamydia incidence among gay, bisexual and other men who have sex with men (gbMSM) and is included in US and UK guidelines.
- Elsewhere, adoption has been cautious due to concerns about antimicrobial resistance, and community-level data remain limited.

**WHAT THIS STUDY ADDS**

- This first survey in Ireland shows that more than one-quarter of gbMSM participants already use DoxyPEP, with high awareness and strong interest in wider adoption.
- Use is concentrated among gbMSM with prior HIV PEP use and those reporting higher sexual activity and sexually transmitted infection history.

**HOW THIS STUDY MIGHT AFFECT RESEARCH, PRACTICE OR POLICY**

- These findings underline the need for internationally aligned, evidence-based policies on DoxyPEP that balance community demand with antimicrobial stewardship.
- Structured, clinician-led access will be essential to ensure safe, equitable and effective implementation across diverse settings.

## Introduction

 Bacterial sexually transmitted infections (STIs) such as syphilis, gonorrhoea and chlamydia continue to pose a significant public health challenge in Ireland. While the Health Protection Surveillance Centre reported an 11% decrease in STI notifications in 2024 compared with 2023, overall rates remain 31% higher than in 2019, the year preceding the COVID-19 pandemic.^1^ This indicates a sustained and elevated burden of STIs.

In most high-income countries, public health response to STIs has generally centred on ‘test and treat’ models of care, which rely on regular screening, prompt diagnosis and timely treatment.^2–4^ While these strategies remain a common approach for reducing transmission, they have not been successful in reversing the upward trend in prevalence among key populations, including gay and bisexual men and other men who have sex with men (gbMSM). This has prompted consideration of additional prevention measures that could complement existing approaches and address the disproportionate burden of bacterial STIs in these groups.

One such measure is the use of antibiotics as a preventive tool to reduce bacterial STI transmission, often referred to as antimicrobial prophylaxis. Doxycycline, a broad-spectrum tetracycline-class antibiotic, is a first-line treatment for chlamydia and a second-line treatment for syphilis, but not for gonorrhoea. More recently, doxycycline has been investigated for its potential as post-exposure prophylaxis (PEP) or pre-exposure prophylaxis (PrEP) to prevent bacterial STIs, particularly among gbMSM and transgender women.

Randomised clinical trials have demonstrated that doxycycline, when taken shortly after condomless sexual activity, can substantially reduce the incidence of bacterial STIs, particularly syphilis and chlamydia.^5–8^ This evidence has informed policy in some jurisdictions. For example, the US Centers for Disease Control and Prevention (CDC) incorporated doxycycline PEP (DoxyPEP) into its 2024 guidelines for STI prevention among high-risk populations.^9^

Studies exploring both daily and event-driven doxycycline strategies as STI PEP and PrEP^10 11^ report high levels of acceptability among gay and bisexual men already familiar with biomedical prevention such as HIV PrEP. DoxyPEP is perceived as empowering and beneficial to sexual well-being, offering ‘peace of mind’ and a sense of control over sexual health.^12 13^ At the same time, concerns persist around antibiotic resistance and potential stigma, which may influence uptake.^11 14^

In Europe, implementation has been cautious due to concerns about long-term safety, ecological impact and antimicrobial resistance.^15^ Current recommendations restrict doxycycline prophylaxis to research settings or selected individuals under specialist care, with strong emphasis on surveillance and stewardship.^16 17^ In contrast, recent UK British Association for Sexual Health and HIV guidelines recommend considering DoxyPEP for gbMSM and other groups at increased STI risk, including transgender people and sex workers.^18^

Community-based studies in the USA and Australia suggest strong willingness to use DoxyPEP, particularly when clear clinical guidance is available,^19 20^ underscoring the importance of timely policy development in other contexts such as Ireland.

In Ireland, there are currently no formal guidelines for doxycycline prophylaxis; however, interim guidelines and follow-up community guidance have been developed in anticipation of formal guideline development.^21^ These interim guidelines summarise the current evidence and provide dosage advice for doxycycline prophylaxis, but they do not constitute a formal recommendation for widespread clinical adoption.

Furthermore, research conducted during the 2022 mpox outbreak found that the gbMSM community was highly engaged with sexual health information and responsive to public health interventions, with rapid uptake of vaccination and other protective measures.^22^ Research on the experiences of gbMSM during the COVID-19 outbreaks also indicates significant compliance with public health measures.^23^ This indicates a potential readiness to consider innovative approaches such as DoxyPEP.

Against a backdrop of rising STI rates, emerging evidence and divergent policy approaches, there is a pressing need to understand awareness, attitudes and patterns of DoxyPEP use in Ireland, where no such data exist. This study reports findings from a 2025 cross-sectional survey to assess factors associated with DoxyPEP use among gbMSM, and to inform clinical practice, public health strategies and policy development.

## Methods

Between May and June 2025, we conducted an anonymous, cross-sectional online survey to explore awareness, use, attitudes and concerns regarding DoxyPEP and other STI prevention strategies among gbMSM in Ireland. The questionnaire was adapted for cultural specificity from a similar study conducted in the USA.^24^ Adaptations were informed by consultation with Irish gbMSM community members to ensure linguistic and contextual relevance such as within the demographics section and references to health system (sources of information and sources of antibiotics).

Eligibility criteria and targeted recruitment included being aged 18 years or older, identifying as male (cisgender or transgender) or as non-binary/gender diverse, and having had sexual contact with a man in the past 12 months. A small number of women also participated through self-selection but were excluded from the analysis.

Recruitment took place through targeted social media posts and lesbian, gay, bisexual, transgender, queer and other sexual and gender minority community organisations. The survey, hosted on SurveyMonkey, included digital informed consent and an Internet Protocol restriction to prevent duplicate entries. It took 10–15 min to complete and no incentives or compensation were offered.

The questionnaire comprised five sections (online supplemental file 1). Section 1 captured demographic information, including gender identity, sexual orientation, age, ethnicity, education, disability and income. Section 2 examined HIV and STI history, including HIV status, HIV PrEP/PEP awareness and use and STI diagnoses within the past 12 months. Section 3 examined sexual behaviour in the previous year, including partner number, positioning, condom use, group sex, chemsex and attendance at sex-on-premises venues. Section 4 assessed awareness and use of antibiotics for STI prevention, including information sources, access, dosing, targeted infections and perceived effects on condom use. Section 5 included Likert-scale attitudinal items on perceived risk, health impact and the importance of STI prevention.

Analyses were prespecified prior to data collection and participants with incomplete responses were excluded using listwise deletion to ensure consistency across analyses. Data were exported from SurveyMonkey to IBM SPSS Statistics (V.29.0; IBM) for analysis. Descriptive statistics were used to summarise participant demographics, behaviours, prophylaxis awareness and use patterns. Univariable logistic regression models were used to examine associations of self-reported DoxyPEP use in the past 12 months with demographic, behavioural and clinical factors. Variables significant at p<0.05 in univariable analysis were included in multivariable logistic regression models using backward selection. These included HIV PEP use, lifetime STI diagnosis, >10 sexual partners and group sex in the past 12 months. The multivariable model demonstrated acceptable fit (Hosmer-Lemeshow χ²(6)=3.03, df=6, p=0.81).

## Results

There were a total of 172 participants that responded to the survey. When incomplete responses (n=21) and cisgender women respondents (n=2) were removed, we had a sample of 149 participants.

### Participant characteristics

Participants ranged in age from 22 to 67 years, the mean age was 36.4 years (SD 7.70), and the median age was 35 years (IQR 25–45). Most participants identified as cisgender men (n=138, 92.6%), with a small number identifying as transgender men, non-binary/genderqueer or another gender identity. In terms of sexual orientation, the majority identified as gay/lesbian/homosexual (n=129, 86.6%), followed by bisexual/pansexual (n=20, 13.4%).

Most respondents were white (n=126, 84.6%), reporting high educational attainment with degree or higher (n=123, 82.6%). Disability status comprised yes (n=13, 8.7%), no (n=135, 90.6%) and prefer not to say (n=1, 0.7%). There was a broad distribution of income, with most participants earning between €20 000 and €79 999 annually (n=112, 74.7%). Smaller proportions reported incomes of €80 000 or more (n=27, 18.1%) or below €20 000 (n=10, 6.7%). Most participants resided in Dublin (n=116, 77.9%).

Regarding HIV status, 13 participants (8.84%) were living with HIV. Awareness of HIV PrEP was universal (n=149, 100%). 103 (69.1%) participants reported currently using HIV PrEP, while 49 (32.9%) reported ever using HIV PEP. Lifetime STI diagnosis was common (n=123, 82.6%); 60 participants (40.2%) reported an STI in the previous 12 months and 25 (16.8%) reported multiple STIs in the previous 12 months (table 1). A full outline of participant characteristics is available in online supplemental table S1.

**Table 1:** Sample of participant characteristics

Variable	N=149	%
Gender identity		
Cisgender men	138	92.6
Transgender men	5	3.4
Non-binary/genderqueer (AMAB)/Transgender women/Other	6	4.0
Sexual orientation		
Gay/Lesbian/Homosexual	129	86.6
Bisexual/Pansexual	20	13.4
Age (years)		
18–24	7	4.7
25–34	60	40.3
35–44	65	43.6
45–54	13	8.7
55–67	4	2.7
Region of residence		
Dublin	116	77.9
Connaught	14	9.4
Munster	7	4.7
Leinster (outside of Dublin)	6	4
Ulster	6	4
Ethnicity		
White	126	84.6
Latin American	13	8.7
Other ethnicities	10	6.7
Education		
Degree or higher	123	82.6
Higher education<degree	18	12.1
Completed secondary school	7	4.7
Other education	1	0.7
HIV status		
Living with HIV	13	8.7
HIV negative	134	90
Never taken an HIV test	2	1.3
HIV PrEP awareness		
Aware of HIV PrEP	149	100
HIV PrEP/PEP use		
Ever used HIV PrEP	113	75.8
Current HIV PrEP use	103	69.1
Ever used HIV PEP	49	32.9
STI diagnosis		
Lifetime STI diagnosis	123	82.6
STI diagnosis in previous 12 months	60	40.3
Multiple STIs in previous 12 months	25	16.8

AMAB, Assigned Male at Birth; PEP, postexposure prophylaxis; PrEP, pre-exposure prophylaxis; STI, sexually transmitted infection.

### Sexual behaviour and STI attitudes

In the past 3 months, half of participants (n=73, 49.0%) reported two to nine anal/frontal sexual partners, with almost one-fifth reporting >10 (n=34, 22.8%). For oral sex, more than half reported two to nine partners (n=82, 55.0%) and 28.9% (n=43) reported >10 partners. Regarding sexual positioning, nearly half (n=69, 46.3%) engaged in both insertive and receptive anal/frontal sex, while 26.2% (n=39) were receptive-only and 22.8% (n=34) were insertive-only. Condom use with casual partners was low overall, with 39.6% (n=59) reporting they never used condoms and a further 29.5% (n=44) reporting rare use.

Group sex in the past 12 months was reported by 64.4% (n=96), most commonly a few times (n=55, 36.9%) or once (n=30, 20.1%). Chemsex was less frequently reported, with 69.1% (n=103) never engaging, although 22.2% (n=33) reported doing so a few times. Attendance at sex-on-premises venues was common, with 38.3% (n=57) attending a few times and 13.4% (n=20) once in the past year.

Most participants expressed concern about STIs, with three-quarters (n=114, 76.5%) agreeing or strongly agreeing that they worry about getting an STI, and over half (n=82, 55%) agreeing it could seriously affect their health. Views on severity varied, with just under one-third (n=49, 32.9%) agreeing that ‘getting an STI is no big deal’, while 62 (41.6%) disagreed. Perceived likelihood was generally high: 88 (59%) disagreed or strongly disagreed that they were unlikely to get an STI, and 115 (77.2%) could not picture themselves avoiding one entirely. Prevention was prioritised, with nearly nine in 10 (n=131, 87.9%) agreeing or strongly agreeing it was important to avoid STIs. Salience was more mixed, with about half (n=74, 49.7%) agreeing they often think about STIs (table 2). A full outline of sexual behaviour and attitudes is available in online supplemental table S2.

**Table 2:** Sexual behaviour and STI attitudes

Variable	N=149	%
Anal/Frontal sexual partners (past 3 months)		
0–1	42	28.2
>10	34	22.8
Oral sexual partners (past 3 months)		
0–1	24	16.1
>10	43	28.9
Anal/Frontal sexual positions (past 3 months)		
Receptive-only	39	26.2
Insertive-only	34	22.8
Both insertive/receptive	69	46.3
Oral-only	7	4.7
Condom use with casual partners (past 3 months)		
Always	9	6.0
Rarely	44	29.5
Never	59	39.6
Group sex (past 12 months)		
Never	53	35.6
Once	30	20.1
More than once	66	44.3
Chemsex (past 12 months)		
Never	103	69.1
Once	7	4.7
More than once	39	26.2
Sex on premises venue use (past 12 months)		
Never	60	40.3
Once	20	13.4
More than once	69	46.3
I worry about getting an STI		
Strongly agree	47	31.5
Agree	67	45
Neither agree nor disagree	18	12.1
Disagree	13	8.7
Strongly disagree	4	2.7
Getting an STI is no big deal		
Strongly agree	10	6.7
Agree	39	26.2
Neither agree nor disagree	38	25.5
Disagree	44	29.5
Strongly disagree	18	12.1
I feel I am unlikely to get an STI		
Strongly agree	10	6.7
Agree	15	10.1
Neither agree nor disagree	36	24.1
Disagree	53	35.6
Strongly disagree	35	23.5
It is important to me to avoid STIs		
Strongly agree	59	39.6
Agree	72	48.3
Neither agree nor disagree	16	10.7
Disagree	1	0.7
Strongly disagree	1	0.7

STI, sexually transmitted infection.

### Awareness, attitudes and use of antibiotic STI prophylaxis

Awareness of using antibiotics as STI prophylaxis was high, with 124 participants (83.2%) reporting awareness. Interest in use was also substantial, with 103 participants (69.1%) indicating they were extremely interested in taking antibiotic STI prophylaxis if it were available. All of those who were previously unaware of antibiotic STI prophylaxis (n=25) reported interest in using the intervention if available, with 14 (56%) extremely interested. Reported sources of information included friends (n=67, 54%), social media (n=59, 47.6%), community organisations (n=44, 35.5%), sexual health clinicians (n=35, 28.2%), online groups (n=35, 28.2%), sexual partners (n=24, 19.4%), news outlets (n=21, 16.9%), other clinicians (n=7, 5.7%) and other sources (n=6, 4.8%).

The most commonly reported concern about using antibiotics to prevent STIs was antimicrobial resistance, reported by 104 participants (69.8%). Other common concerns included the inconvenience of using antibiotics around sex (n=87, 58.4%), cost (n=61, 40.9%) and long-term side effects (n=58, 38.9%). Concerns were also raised about short-term side effects (n=45, 30.2%), adherence (n=16, 10.7%), lack of information (n=15, 10.1%) and privacy (n=13, 8.7%). A smaller proportion indicated concerns such as not being at risk for STIs (n=12, 8.1%) or listed other reasons (n=7, 4.7%), including the need to attend a doctor, concerns about misuse by others, confusion around dosing and repeated use impacting effectiveness.

Use of antibiotic STI prophylaxis was reported by 44 participants (29.5%) for lifetime use and 42 (28.2%) for use in the past 12 months. Among those who had used antibiotic prophylaxis, the most common antibiotic was doxycycline (n=43, 97.7%), followed by azithromycin (n=5, 11.4%), amoxicillin (n=3, 6.8%), penicillin (n=2, 4.5%), ceftriaxone (n=2, 4.5%) and erythromycin (n=1, 2.3%). Sources of antibiotics included sexual health clinics (n=18, 40.9%), online (prescribed; n=12, 27.3%), friends or partners (n=11, 25%), online (not prescribed; n=10, 22.7%), non-sexual health clinics (n=4, 9.1%), leftover antibiotics from a non-STI condition (n=4, 9%), other sources (n=7, 15.9%) and leftover antibiotics from an STI (n=1, 2.3%).

Among those who reported DoxyPEP use in the past 12 months, the majority (n=41, 97.6%) had taken the recommended 200 mg dosage. Reported reasons for use included chlamydia (n=32, 72.7%), gonorrhoea (n=31, 70.5%), syphilis (n=27, 61.4%) and general STI prevention (n=22, 50%), with one participant (2.3%) citing other reasons. Most participants (n=36, 81.8%) reported no change in condom use while taking antibiotic STI prophylaxis, five noted condom use decreased (11.4%) and three stated condom use increased (6.8%) (table 3). A full outline of sexual behaviour and attitudes is available in online supplemental table S3.

**Table 3:** Awareness, attitudes and use of antibiotic STI prophylaxis

Variable	N	%
Awareness of antibiotic STI prophylaxis	149	
Awareness of antibiotic STI prophylaxis	124	83.2
Source of information on antibiotic STI prophylaxis*	124	
Friend	67	54
Social media	59	47.6
Online community group	44	35.5
Sexual health clinician	35	28.2
Community organisation	35	28.2
Sexual partner	24	19.4
News outlet	21	16.9
Other clinician	7	5.7
Interest in antibiotic STI prophylaxis		
Extremely interested in using prophylaxis	103	69.1
Moderately interested in antibiotic STI prophylaxis	21	14.1
Slightly interested in antibiotic STI prophylaxis	19	12.8
Not interested at all	6	4
Concerns about antibiotic STI prophylaxis*	149	
Costs of antibiotics	61	40.9
Inconvenience	87	58.4
Adherence	16	10.7
Privacy	13	8.7
Side effects	103	70.1
Antibiotic resistance	104	69.8
Lack of information	15	10.1
Not having enough risk	12	8.1
Antibiotic STI prophylaxis use	149	
Ever used antibiotic prophylaxis	44	29.5
Used antibiotic prophylaxis past 12 m	42	28.2
Antibiotics used*†	44	
Doxycycline	43	97.7
Other antibiotic	13	29.9
Sources of antibiotics*†	44	
Sexual health clinic	18	40.9
Non-sexual health clinic	4	9.1
Online (prescribed/non-prescribed)	22	40
Leftover antibiotics	5	11.4
Friend/Partner	11	25
STIs aiming to avoid with antibiotic STI prophylaxis*†	44	
Chlamydia	32	72.7
Gonorrhoea	31	70.5
Syphilis	27	61.4
All STIs	22	59
HIV PrEP use with STI prophylaxis†	44	
STI prophylaxis use on HIV PrEP	38	86.4
Condom use change (while on antibiotic STI prophylaxis)†	44	
Condom use decreased	5	11.4
Condom use unchanged	36	81.8
Dosing of DoxyPEP‡	42	
200 mg (recommended)	41	97.6
Unsure	1	2.4

* Participants may select more than one option.

† Only those who reported antibiotic STI prophylaxis.

‡ Only those who reported DoxyPEP use.

DoxyPEP, doxycycline postexposure prophylaxis; PrEP, pre-exposure prophylaxis; STI, sexually transmitted infection.

### Predictors of antibiotic prophylaxis use

Logistic regression models were used to explore predictors of antibiotic STI prophylaxis use among survey participants (table 4). Model summary statistics showed a −2 log likelihood of 154.987, with Cox and Snell R²=0.159 and Nagelkerke R²=0.227, suggesting that approximately 16%–23% of the variance in prophylaxis use was explained by the included variables. Multicollinearity was assessed using collinearity diagnostics in SPSS 29, including tolerance, variance inflation factor, condition indices and variance proportions. No evidence of problematic multicollinearity was identified.

**Table 4:** Logistical regressions: predictors of antibiotic STI prophylaxis use

Variable	COR	95% CI	P value	AOR	95% CI	P value
Age	1.47	0.951 to 2.27	0.83			
HIV-positive status	2.14	0.68 to 3.68	0.195			
Current HIV PrEP use	2.53	0.97 to 6.61	0.058			
HIV PEP use	3.33	1.59 to 6.97	0.001	3.019	1.35 to 6.73	0.007
Ever been diagnosed with an STI	6.22	1.40 to 27.61	0.016	4.86	1.04 to 22.78	0.45
STI diagnosis in past 12 months	1.08	0.51 to 2.27	0.846			
Multiple STI	1.08	0.37 to 3.16	0.891			
>10 lifetime sexual partners	2.76	1.25 to 6.18	0.012	1.20	0.48 to 3.01	0.70
Sex on premises attendance	1.671	0.795 to 3.510	0.175			
Chemsex	1.180	0.558 to 2.496	0.665			
Group sex	3.65	1.55 to 8.59	0.003	3.28	1.26 to 8.51	0.02

AOR, adjusted OR; COR, crude OR; PEP, postexposure prophylaxis; PrEP, pre-exposure prophylaxis; STI, sexually transmitted infection.

In univariable analyses, predictors associated with antibiotic prophylaxis use included HIV PEP use (crude OR (COR) 3.33; 95% CI 1.59 to 6.97; p=0.001), group sex (COR 3.65; 95% CI 1.55 to 8.59; p=0.003), >10 sexual partners in the past year (COR 2.76; 95% CI 1.25 to 6.13; p=0.012) and lifetime STI diagnosis (COR 6.22; 95% CI 1.40 to 27.61; p=0.016; table 4).

Other variables, including age, HIV-positive status, current HIV PrEP use, recent (12-month) STI diagnosis, multiple STI diagnoses, chemsex participation, and sex-on-premises venue attendance, were not significantly associated with prophylaxis use in univariable models.

In the multivariable model, factors that remained associated with antibiotic prophylaxis use included HIV PEP use (adjusted OR (AOR) 3.02; 95% CI 1.35 to 6.73; p=0.007), group sex (AOR 3.28; 95% CI 1.26 to 8.51; p=0.015) and lifetime STI diagnosis (AOR 4.86; 95% CI 1.04 to 22.78; p=0.045), although the wide CI suggests some uncertainty in the estimate. In contrast, the association between antibiotic prophylaxis use and having >ten sexual partners (AOR 1.20; 95% CI 0.48 to 3.01; p=0.697) was no longer statistically significant after adjustment.

### Discussion

This study provides insights into the early use of DoxyPEP for STI prevention among gbMSM in Ireland. Although international evidence and policy developments have moved rapidly in this area, our findings suggest that Ireland is still in the early stages of community uptake, with no current clinical guidelines in place. Nonetheless, the fact that over one-quarter of respondents reported DoxyPEP use within the past year, and more than two-thirds of respondents reported interest, highlights a growing need to consider biomedical strategies for STI prevention, even in the absence of formal policy or structured access.

A critical issue emerging from this study is the informal sourcing of doxycycline. Nearly half of users accessed antibiotics outside formal healthcare settings, including from friends, online vendors and leftover prescriptions. This mirrors concerns raised in other countries and heightens the urgency for policy intervention. Unregulated use raises the risk of inappropriate dosing, use of non-recommended antibiotics and increased antimicrobial resistance.^15 17 25 26^ These risks are particularly concerning given the documented rise in tetracycline resistance in *Neisseria gonorrhoeae* in Europe and the cautious stance taken by many European health bodies.^16 27^ Although unregulated access raises concerns, nearly all users in this study (97.6%) adhered to correct dosing. It is notable that for the participants in this study, antimicrobial resistance was a prime concern.

Consistent with global evidence that antibiotic STI prophylaxis is chiefly adopted by individuals already highly engaged in sexual health practices, and those reporting higher partner numbers and group sex,^6 10 18–20 28^ qualitative studies also show that this group views DoxyPEP as an extension of existing prevention strategies, balancing perceived benefits with concerns about antimicrobial resistance and stigma.^12 13^ Our findings mirror this pattern: DoxyPEP users were more likely to report prior STIs, group sex, higher partner numbers and previous HIV PEP use, aligning with international prescribing guidance.^9 18^ These associations were evident in both univariable and multivariable models (although a higher number of sexual partners was only associated with the outcome in univariable models), indicating that DoxyPEP is already being used in a targeted way by those at elevated risk of STIs.

Unlike early HIV PrEP implementation studies, where modest behavioural risk compensation was associated with HIV PrEP use, such as a reduction in condom use,^29 30^ the majority of DoxyPEP users in this study (81.8%) reported no change in condom use, suggesting that antibiotic prophylaxis adoption may not be accompanied by similar shifts in perceived risk or preventive behaviour. STIs remained a significant concern for participants, with most worrying about acquiring an STI and valuing avoidance. The small overlap in some responses, where individuals both agree or strongly agree that they worry about getting an STI and also agree that ‘getting an STI is no big deal’, suggests some attitudinal ambivalence. Participants may worry about STIs yet still view them as generally manageable. This context may help explain the strong interest in DoxyPEP.

Lessons from the rapid and community-driven response to the 2022 mpox outbreak in Ireland, which included high uptake of vaccination and sexual health measures, indicate that gbMSM are responsive to innovation when trusted and evidence-informed services are in place.^22^ This foundation can support the introduction of structured, clinician-led DoxyPEP services, particularly within existing HIV PrEP programmes.

However, implementation must be accompanied by clear prescribing guidelines, clinician education, behavioural support and antimicrobial stewardship. Additionally, equity must be prioritised. Our findings highlight high awareness and interest in DoxyPEP among participants already engaged with HIV prevention and sexual health services. However, this aligns with broader concerns that current research and access models remain centred on cisgender gay and bisexual men in high-income urban settings. Future implementation must address equity from the outset, ensuring inclusive design for those most affected by structural barriers, including trans people, people with vaginas and communities in lower-resource or rural settings.^31^

### Limitations

The small sample limits statistical power and generalisability. Recruitment was not incentivised, and ethical approval restricted us to public-only recruitment rather than purposive sampling in clinical settings. The sample was predominantly white, highly educated and urban-based, reflecting online and community-based recruitment, and may not represent migrant or socioeconomically marginalised gbMSM. Respondents were mostly based in Dublin, further limiting generalisability to rural populations. The absence of reliable census data on the size and demographics of the gbMSM population in Ireland also made it difficult to determine a representative sample or calculate statistical power. These limitations highlight the need for more inclusive sampling strategies and stronger data infrastructure to support equitable STI prevention policy.

## Conclusion

In summary, from a public health perspective, Ireland is well placed to respond to growing STI rates among gbMSM with DoxyPEP, because there is already a high level of awareness, community interest and targeted informal use of DoxyPEP among those who may be at increased risk. This early engagement, combined with the proven responsiveness of gbMSM to evidence-based interventions and a strong community organisation infrastructure, provides a strong foundation for the introduction of structured, equitable and clinically guided DoxyPEP services. Concerns around antimicrobial resistance highlight the need for clear guidance and surveillance as DoxyPEP use evolves. This duality, enthusiastic community uptake alongside apprehension about antimicrobial resistance, echoes findings from the USA and Australia, where participants emphasised the need for accurate information, public health messaging and clinician support to ensure safe use.^12 14 19^ To ensure safety, effectiveness and equity, there is now an urgent need for national policy that is evidence-based, community-informed and embedded within holistic sexual health services.

## Supplementary material

10.1136/sextrans-2025-056745 online supplemental table 1

10.1136/sextrans-2025-056745 online supplemental table 2

10.1136/sextrans-2025-056745 online supplemental table 3

10.1136/sextrans-2025-056745 online supplemental file 1

## Data Availability

Data are available on reasonable request.
