# Supplementary material for: Awareness, attitudes and early use of doxycycline prophylaxis among gbMSM in Ireland: findings from a 2025 community-based cross-sectional survey
Source: Sex Transm Infect. 2026 Jan 14;102(6):e056745. doi: 10.1136/sextrans-2025-056745 (PMC13539834; doi:10.1136/sextrans-2025-056745)
Supplement: Supplementary file 1 [file sextrans-102-6-s004.pdf]

DoxyPEP Ireland Survey

Participant Information and Consent form

* 1. **What is this research about?**

This research study explores awareness, interest, and use of doxycycline post-exposure prophylaxis (DoxyPEP) and other antibiotics for preventing bacterial sexually transmitted infections (STIs) among gay, bisexual, and other men who have sex with men (gbMSM) and trans people in Ireland.

About us

Our research team is made up of healthcare professionals and researchers with expertise in sexual health and public health.
The research team includes
Dr John P Gilmore
Mr David J Field
Dr Chris Noone
Dr Michael Traeger
Dr John White

Why are we doing this research?

There is growing interest in the use of antibiotics to prevent STIs, particularly DoxyPEP. However, little is known about how many people are using STI prophylaxis, how they are accessing it, and their understanding of its benefits and risks. This study aims to gather data to inform public health strategies and sexual health services in Ireland.

Why are we asking you to take part in this study?

If you are aged 18 or older and live in Ireland, your experiences and opinions on STI prevention strategies, including antibiotic use, are valuable. Your participation will help us understand how widely doxyPEP and other antibiotics are being used and inform guidance for safe and effective STI prevention.

Why might you want to take part in this study?

By participating, you will contribute to research that could improve sexual health services and public health recommendations. Your insights will help shape education, policy, and clinical practice around STI prevention in Ireland.

What will happen if you decide to take part in this research study?

If you choose to participate, you will complete an anonymous online survey hosted on SurveyMonkey. The survey consists of approximately 32 questions and should take no more than 15 minutes to complete. Most questions will ask about your awareness and use of STI prophylaxis, as well as some information on your sexual health behaviours.

How will your data be used?

Your participation in this study is completely anonymous. No identifiable information will be collected. The responses will be analysed as a group to provide insights into STI prophylaxis use in Ireland. The findings may be published in academic journals, reports, and presentations, but no individual responses will be identifiable.

How will your privacy be protected?

Your privacy is a top priority. All data will be securely stored and only accessible to the research team. As the survey is completely anonymous, there is no way for responses to be linked back to individuals.

What are the benefits of taking part in this research study?

By participating, you will contribute to important research that may lead to better sexual health policies and services in Ireland. Your input will help ensure that STI prevention strategies are informed by real-world experiences.

What are the potential risks of taking part in this research study?

The risks of participating are minimal. Some participants may find certain survey questions sensitive, as they relate to sexual health practices.

If you need support as a result of your participation in this survey you can contact any of the organisations or helplines below

LGBT Ireland Helpline 1890 929539 or online chat at lgbt.ie
The Switchboard (01) 872 1055
Samaritans 116 123

Can you change your mind at any stage and withdraw from the study?

Participation in this study is entirely voluntary. However, once you submit the survey, it will not be possible to withdraw your responses, as all data is collected anonymously and cannot be linked back to individual partic

- [ ] I understand and consent that I have had the opportunity to ask questions and discuss the study if I want further information. (Note you can contact john.gilmore@ucd.ie).
- [ ] I understand and consent that I have received satisfactory answers to all my questions, if I have had a query.
- [ ] I understand and consent that I have received enough information about this study.
- [ ] I understand and consent that my responses in the online survey will be downloaded onto a university server and stored securely
- [ ] I understand and consent that I will not be able to withdraw my answers once submitted as the data is anonymised
- [ ] I understand and consent that anonymised data will be archived for 10 years for future research and then destroyed
- [ ] I agree to take part in the study.

☐ I understand and consent that I have read and understood the attached Participant Information for this study.

Demographics

We would like to ask about your personal background.

* 2. What is your age?

* 3. What is your gender identity?

- Cisgender man (cisgender means your gender identity matches the sex you were assigned at birth).
- Cisgender woman (cisgender means your gender identity matches the sex you were assigned at birth).
- Transgender man
- Transgender woman
- Nonbinary/genderqueer (assigned male sex at birth)
- Nonbinary/genderqueer (assigned female sex at birth)
- Other (please specify)

* 4. What is your sexual orientation?

- Gay/lesbian/homosexual
- Bisexual/pansexual
- Heterosexual
- Other (please specify)

* 5. What is your racial/ethnic background?

- White
- Black
- Asian
- Irish Traveller
- Latin American
- Mixed/Multiracial
- Other (please specify)

* 6. What is the highest level of education you have completed?

- ◯ Degree or higher degree
- ◯ Higer Education below degree (ie Diploma, Higher Certificate)
- ◯ Leaving certificate or equivilent
- ◯ Junior/Intermediate/group certificate or equivilent
- ◯ No educational qualifications
- ◯ Other (please specify)

[ ]

* 7. What is your approximate annual income (EUR)?

- ◯ 0-9,999
- ◯ 10,000-19,999
- ◯ €20,000–€39,999
- ◯ €40,000–€59,999
- ◯ €60,000–€79,999
- ◯ €80,000 or more

* 8. Do you have a Disability

- ◯ Yes
- ◯ No
- ◯ Prefer not to say

* 9. In which county do you reside?

- Carlow
- Cavan
- Clare
- Cork
- Donegal
- Dublin
- Galway
- Kerry
- Kildare
- Kilkenny
- Laois
- Leitrim
- Limerick
- Longford
- Louth
- Mayo
- Meath
- Monaghan
- Offaly
- Roscommon
- Sligo
- Tipperary
- Waterford
- Westmeath
- Wexford
- Wicklow

HIV testing

* 10. When was your most recent HIV test?

- ○ less than 6 months ago
- ○ between 6 months and 1 year ago
- ○ between 1 and 5 years ago
- ○ more than 5 years ago
- ○ I've never tested for HIV

HIV status

* 11. Do you currently live with HIV?

- ◯ Yes
- ◯ No
- ◯ Prefer not to say

HIV treatment

* 12. Are you currently on treatment for HIV?

- Yes
- No
- Prefer not to say

HIV PrEP Awareness

HIV PrEP is a medication that someone who is HIV-negative can take before having sex to protect themselves from getting HIV.

* 13. Have you heard of HIV PrEP before taking this survey?

- ◯ Yes
- ◯ No

HIV PrEP Use

HIV PrEP is a medication that someone who is HIV-negative can take before having sex to protect themselves from getting HIV.

* 14. Have you ever used HIV PrEP?

- ◯ Yes
- ◯ No

Current HIV PrEP Use

* 15. Are you currently using HIV PrEP?

- ○ Yes
- ○ No

* 16. In the past 12 months how have you used PrEP (please select all that apply)

- ☐ I haven’t used HIV PrEP in the past 12 months
- ☐ Daily (taking pills every day)
- ☐ Event-based (2-1-1)
- ☐ Other (please specify)

* 17. Where have you sourced you HIV PrEP (Check all that apply)

- ☐ From a public STI service
- ☐ From a private STI service
- ☐ From a General Practitioner (GP)
- ☐ Bought it online
- ☐ Got from partners/friends
- ☐ Other (please specify)

HIV PEP

HIV post-exposure prophylaxis (PEP) is a medication that someone who is HIV-negative can take after having sex to protect themselves from getting HIV.

* 18. Have you ever used PEP for HIV?

- ◯ Yes
- ◯ No
- ◯ Unsure/Prefer not to answer

STI diagnosis

Sexually Transmitted Infections (STIs) are specific infections which are transmitted during sexual contact and include Syphillis, Gonorrhea, Genital Herpes, Mpox, Mychoplasma Genitalium, Genital Warts, Lymphogranuloma venereum (LGV).

* 19. Have you ever been diagnosed with an STI?

- ◯ Yes
- ◯ No
- ◯ Prefer not to say

DoxyPEP Ireland Survey

STI Diagnosis over 12 months

Sexually Transmitted Infections (STIs) are specific infections which are transmitted during sexual contact and include Syphillis, Gonorrhea, Genital Herpes, Mpox, Mychoplasma Genitalium, Genital Warts, Lymphogranuloma venereum (LGV).

* 20. Have you been diagnosed with an STI in the past 12 months?

- ◯ Yes
- ◯ No
- ◯ Prefer not to say

* 21. Have you been diagnosed with multiple (more than one) STIs in the past 12 months?

- ◯ Yes
- ◯ No
- ◯ Prefer not to say

Awareness of Antibiotic STI Prophylaxis

The following questions are about experiences you may have had with taking antibiotics to prevent STIs (e.g. chlamydia, gonorrhea or syphilis).

Taking antibiotics around the time of sex to prevent yourself from getting an STI is called STI prophylaxis and may also be called STI PEP or STI PrEP.

This is different from STI treatment, which is the use of antibiotics to treat an STI one already has been diagnosed with.

The only antibiotic currently recommended for this use is Doxycycline, which is taken within 72 hours after sex at a single dose of 200mg. This use is referred to as DoxyPEP

* 22. (Before this survey) Have you ever heard of using antibiotics to prevent STIs (before or after sex)?

- ◯ Yes
- ◯ No

Sources of information on Antibiotic STI prophylaxis

* 23. Where did you hear about taking antibiotics to prevent STIs? (Please select all that apply.)

- [ ] Doctor, nurse or other clinician in a sexual health clinic
- [ ] Doctor, nurse or other clinician in another setting (not sexual health)
- [ ] Online community group (e.g., PrEP Facts Facebook group)
- [ ] Community organisation (e.g., LGBT organisation)
- [ ] Social media
- [ ] Friend
- [ ] Regular or casual sex partner
- [ ] News outlet (e.g., online news site)
- [ ] Other (please specify)

Interest in STI prophylaxis

* 24. If available would you be interested in using antibiotics around the time of sex for STI prevention?

- ◯ Not at all interested
- ◯ Slightly interested
- ◯ Moderately interested
- ◯ Extremely intersted

* 25. Would you have any of the following concerns about taking antibiotics to prevent STIs?

- ☐ Cost of antibiotics
- ☐ Inconvenience of getting a prescription
- ☐ Not remembering to take the medicine around the time of sex
- ☐ Not wanting people to know I am taking antibiotics to prevent STIs
- ☐ Short term side-effects
- ☐ Long-term side-effects
- ☐ Worried about antibiotic resistance
- ☐ Not enough information to decide if I should use it
- ☐ Not enough STI risk to need it
- ☐ Other (please specify)

[ ]

Getting information on antibiotic STI prophylaxis

* 26. How would you prefer to get information about taking antibiotics to prevent STIs? (Please select all that apply.)

- [ ] Doctor, nurse or other clinician in a sexual health clinic
- [ ] Doctor, nurse or other clinician in another setting (not sexual health)
- [ ] Community organisation (e.g., local community group)
- [ ] Social media
- [ ] Friend
- [ ] Regular or casual sex partner
- [ ] News outlet (e.g., online news site)
- [ ] Other (please specify)

Taking antibiotic STI prophylaxis

* 27. Have you ever taken antibiotics around the time of sex to prevent an STI?

- Yes
- No

Taking antibiotic STI prophylaxis in 12 months

* 28. Have you taken antibiotics around the time of sex to prevent STIs in the past 12 months? (Please select all that apply.)

- ◯ Yes
- ◯ No

Specific antibiotic use

* 29. Which antibiotics have you ever taken around the time of sex to prevent STIs? (Please select all that apply.)

- [ ] Doxycycline (DoxyPEP)
- [ ] Azithromycin
- [ ] Erythromycin
- [ ] Amoxicillin
- [ ] Gentamicin
- [ ] Ceftriaxone
- [ ] Penicillin
- [ ] Not sure
- [ ] Other (please specify)

Which STIs do you want to prevent

* 30. When taking antibiotics around the time of sex to prevent STIs, which STIs were you trying to prevent? (Please select all that apply.)

- [ ] Chlamydia
- [ ] Gonorrhea
- [ ] Syphilis
- [ ] Any STI
- [ ] Other (please specify)

* 31. When you have used antibiotics around the time of sex to prevent STIs, where did you get the antibiotics? (Please select all that apply.)

- [ ] A doctor, nurse or other clinician in a sexual health clinic prescribed them to me to prevent STIs
- [ ] Doctor, nurse or other clinician in another setting (not sexual health) prescribed them to me
- [ ] I bought them online with a prescription
- [ ] I bought them online without a prescription
- [ ] I had leftover antibiotics from treatment of a previous STI
- [ ] I had leftover antibiotics that I was prescribed for something else
- [ ] A friend or partner gave them to me
- [ ] Other (please specify)

* 32. In the past 12 months, when having sex with casual partners (people who you don't consider your main partner/s or spouse), how often did you take antibiotics around the time of sex to prevent STIs?

- ○ Everytime
- ○ Most of the time
- ○ Some of the time
- ○ Never

DoxyPEP Ireland Survey

Awareness of DoxyPEP

DoxyPEP refers to the use of Doxycycline 200mgs within 72 hours after sex to prevent an STI.

* 33. Before taking this survey, had you heard of DoxyPEP?

- ◯ Yes
- ◯ No

Use of DoxyPEP

What is DoxyPEP?

DoxyPEP stands for doxycycline post-exposure prophylaxis.

Doxycycline is an antibiotic.

Post-exposure prophylaxis is the use of a medical treatment after potential exposure to an infectious agent (bacteria, virus, etc) to prevent an infection from occurring. DoxyPEP is a relatively new way of preventing some sexually transmitted infections (STIs) by using doxycycline after condomless sex, including oral sex.

DoxyPEP refers to the use of Doxycycline 200mgs within 72 hours after sex to prevent an STI.

* 34. Have you used DoxyPEP in the past 12 months?

- ◯ Yes
- ◯ No

Dosage of DoxyPEP

The recommended dosage for DoxyPEP is Doxycycline 200mgs within 72 hours after sex to prevent an STI.

* 35. In the past 12 months, when taking doxycycline after sex to prevent STIs, how many pills did you take and what was the dosage?

- ◯ 1 pill (100mgs)
- ◯ 2 pills (200mg dose)
- ◯ Unsure about dose

DoxyPEP Ireland Survey

Reasons for using antibiotics for STI prevention

The following questions are about reasons you have used antibiotics around the time of sex to prevent STIs. These questions relate to use of any kind of antibiotics.

* 36. Which of the following situations have influenced your decision to use antibiotics to prevent STIs? (Please select all that apply.)

- [ ] Having sex with a casual partner
- [ ] Having sex with a new partner
- [ ] Participating in group sex
- [ ] Having sex with someone other than your regular partner
- [ ] Having sex while travelling
- [ ] Not wanting to get an STI before travelling somewhere
- [ ] Having sex with someone who has told you they have an STI
- [ ] Suspecting that your partner may have an STI
- [ ] Other (please specify)

* 37. Which of the following were reasons you used antibiotics to prevent STIs? (Please select all that apply.)

- [ ] Peace of mind
- [ ] Not wanting to get an STI
- [ ] To feel more comfortable having sex
- [ ] To prevent side effects
- [ ] To prevent passing on an STI to my casual partner(s)
- [ ] To prevent passing on an STI to my regular partner(s)
- [ ] To prevent having to take a different antibiotic if I were diagnosed with an STI
- [ ] To protect my community
- [ ] Suggested by a partner
- [ ] Recommended by a doctor or other clinician
- [ ] Not wanting to use a condom
- [ ] Partner not wanting to use a condom
- [ ] Other (please specify)

* 38. During periods when you were using antibiotics around the time of sex to prevent STIs, did your condom use change?

- Yes, I used condoms more
- Yes, I used condoms less
- No, my condom use stayed the same
- I have not used antibiotics around the time of sex

Potential situations and reasons for using antibiotics

The following questions are about reasons you would be interested in taking antibiotics around the time of sex to prevent STIs.

* 39. In which situations would you be interested in using antibiotics to prevent STIs?

- ☐ Having sex with a casual partner
- ☐ Having sex with a new partner
- ☐ Participating in group sex
- ☐ Having sex with someone other than your regular partner
- ☐ Having sex while travelling
- ☐ Not wanting to get an STI before travelling somewhere
- ☐ Having sex with someone who has told you they have an STI
- ☐ Suspecting that your partner may have an STI
- ☐ Other (please specify)

* 40. Which of the following might be reasons you would be interested in using antibiotics to prevent STIs?

- ☐ Not wanting to get an STI
- ☐ To feel more comfortable having sex
- ☐ To prevent side effects
- ☐ To prevent passing on an STI to my casual partner(s)
- ☐ To prevent passing on an STI to my regular partner(s)
- ☐ To prevent having to take a different antibiotic if I were diagnosed with an STI
- ☐ To protect my community
- ☐ Suggested by a partner
- ☐ Recommended by a doctor or other clinician
- ☐ Not wanting to use a condom
- ☐ Partner not wanting to use a condom
- ☐ Other (please specify)

Antibiotics to treat suspect STIs

The following questions are about experiences you may have had with taking antibiotics to treat a suspected STI that had not been diagnosed by a doctor.

* 41. Have you ever taken antibiotics because you were experiencing STI symptoms?

- ◯ Yes
- ◯ No

* 42. Have you ever taken antibiotics because a recent partner told you they had an STI?

- ◯ Yes
- ◯ No

Sexual beaviour over the last 3 months

* 43. How many anal or frontal/vaginal sex partners have you had **in the past 3 months?**

- 0-1
- 2-9
- 10 or more

* 44. How many oral sex partners have you had **in the past 3 months?**

- 0-1
- 2-9
- 10 or more

* 45. **In the past 3 months**, what has been your primary sexual positioning with casual anal or vaginal/frontal sex partners?

- Receptive (bottom) only
- Insertive (top) only
- Both insertive and receptive
- Only non-penetrative (oral) sex

* 46. How often have you used condoms with casual partners **in the past 3 months?**

- Always
- Very often
- Sometimes
- Rarely
- Never

Sexual beaviour over the last 12 months

* 47. Have you participated in group sex **in the past 12 months?**

- Never
- Once
- A few times
- About once a month or more

* 48. Have you used drugs before or during sex **in the past 12 months** with the intention of prolonging or improving sex (Chemsex).

- Never
- Once
- A few times
- About once a month or more

* 49. Have you had sex in a sauna/bathhouse/darkroom or other sex on premises venue **in the past 12 months?**

- Never
- Once
- A few times
- About once a month or more

* 50. Please rate how much you agree or disagree with the following statements.

	Strongly disagree	Disagree	Neither agree nor disagree	Agree	Strongly agree
I worry about getting an STI	○	○	○	○	○
Getting an STI is something I think about often	○	○	○	○	○
Getting an STI could seriously affect my health	○	○	○	○	○
Getting an STI is no big deal	○	○	○	○	○
I feel I am unlikely to get an STI	○	○	○	○	○
I can't picture myself getting an STI	○	○	○	○	○
It is important to me to avoid STIs	○	○	○	○	○

* 51. Thinking about your sex life during the last 6 months, please rate your satisfaction with the following aspects. If you have had more than one sexual partner in the last 6 months, please respond based on your overall experience.

	Not at all satisfied	A little satisfied	Moderately satisfied	Very satisfied	Extremely satisfied
The intensity of my sexual arousal	○	○	○	○	○
The quality of my orgasms	○	○	○	○	○
My "letting go" and surrender to sexual pleasure during sex	○	○	○	○	○
My focus/concentration during sexual activity	○	○	○	○	○
The way I sexually react to my partner(s)	○	○	○	○	○
My body's sexual functioning	○	○	○	○	○
My emotional opening up in sex	○	○	○	○	○
My mood after sexual activity	○	○	○	○	○
The frequency of my orgasms	○	○	○	○	○
The pleasure I provide to my partner(s)	○	○	○	○	○
The balance between what I give and receive in sex	○	○	○	○	○
My partner(s)' emotional opening up during sex	○	○	○	○	○
My partner(s)' initiation of sexual activity	○	○	○	○	○
My partner(s)' ability to orgasm	○	○	○	○	○
My partner(s)' surrender to sexual pleasure ("letting go")	○	○	○	○	○
The way my partner(s) take care	○	○	○	○	○

of my sexual needs					
My partner(s)' sexual creativity	○	○	○	○	○
My partner(s)' sexual availability	○	○	○	○	○
The variety of my sexual activities	○	○	○	○	○
The frequency of my sexual activity	○	○	○	○	○

Conclusion

Many thanks for taking part in this survey. If you have any follow up questions you can reach out to the prinicpal investigator Dr John Gilmore at john.gilmore@ucd.ie

52. Do you have anything you'd like to add?
