# Supplementary material for: Awareness, attitudes and early use of doxycycline prophylaxis among gbMSM in Ireland: findings from a 2025 community-based cross-sectional survey
Source: Sex Transm Infect. 2026 Jan 14;102(6):e056745. doi: 10.1136/sextrans-2025-056745 (PMC13539834; doi:10.1136/sextrans-2025-056745)
Supplement: Supplementary file 2 [file sextrans-102-6-s001.docx]

# Tables Awareness, Attitudes, and Early Use of Doxycycline Prophylaxis among gbMSM in Ireland: Findings from a 2025 community-based cross-sectional Survey

## Table S1 Participant characteristics

| Variable | n | Overall % |
| --- | --- | --- |
| Overall | 149 |  |
| Gender identity |  |  |
| Cisgender men | 138 | 92.6 |
| Transgender men | 5 | 3.4 |
| Nonbinary/genderqueer (AMAB) | 3 | 2.0 |
| Transgender women | 2 | 1.3 |
| Other gender identity | 1 | 0.7 |
| Sexual orientation |  |  |
| Gay/lesbian/homosexual | 129 | 86.6 |
| Bisexual/pansexual | 20 | 13.4 |
| Age |  |  |
| 18-24 | 7 | 4.7 |
| 25-34 | 60 | 40.3 |
| 35-44 | 65 | 43.6 |
| 45-54 | 13 | 8.7 |
| 55-67 | 4 | 2.7 |
| Region of residence |  |  |
| Dublin | 116 | 77.9 |
| Connaught | 14 | 9.4 |
| Munster | 7 | 4.7 |
| Leinster (Outside of Dublin) | 6 | 4 |
| Ulster | 6 | 4 |
| Ethnicity |  |  |
| White | 126 | 84.6 |
| Latin American | 13 | 8.7 |
| Asian | 4 | 2.7 |
| Mixed/Multiracial | 3 | 2.0 |
| Black | 1 | 0.7 |
| Other ethnicity | 2 | 1.3 |
| Education |  |  |
| Degree or higher | 123 | 82.6 |
| Higher education < degree | 18 | 12.1 |
| Completed secondary school | 7 | 4.7 |
| Other education | 1 | 0.7 |
| Disability status |  |  |
| Yes | 13 | 8.7 |
| No | 135 | 90.6 |
| Prefer not to say | 1 | 0.7 |
| Income |  |  |
| Income €60,000–€79,999 | 41 | 27.5 |
| Income €40,000–€59,999 | 37 | 24.8 |
| Income €20,000–€39,999 | 34 | 23.8 |
| Income €80,000 or more | 27 | 18.2 |
| Income €10,000–€19,999 | 6 | 4.0 |
| Income €0–€9,999 | 4 | 2.7 |
| HIV Status |  |  |
| Living with HIV | 13 | 8.7 |
| HIV negative | 134 | 90 |
| Never taken a HIV test | 2 | 1.3 |
| HIV PrEP Awareness |  |  |
| Aware of HIV PrEP | 149 | 100 |
| HIV PrEP/PEP use |  |  |
| Ever used HIV PrEP | 113 | 75.8 |
| Current HIV PrEP use | 103 | 69.1 |
| Ever used HIV PEP | 49 | 32.9 |
| STI Diagnosis |  |  |
| Lifetime STI diagnosis | 123 | 82.6 |
| STI diagnosis in previous 12 months | 60 | 40.3 |
| Multiple STIs in previous 12 months | 25 | 16.8 |

Table S1 Participant characteristics
