# Supplementary material for: Awareness, attitudes and early use of doxycycline prophylaxis among gbMSM in Ireland: findings from a 2025 community-based cross-sectional survey
Source: Sex Transm Infect. 2026 Jan 14;102(6):e056745. doi: 10.1136/sextrans-2025-056745 (PMC13539834; doi:10.1136/sextrans-2025-056745)
Supplement: Supplementary file 3 [file sextrans-102-6-s002.docx]

# Tables Awareness, Attitudes, and Early Use of Doxycycline Prophylaxis among gbMSM in Ireland: Findings from a 2025 community-based cross-sectional Survey

## Table S2: Sexual behaviour and STI attitudes

| Variable | n | Overall % |
| --- | --- | --- |
| Overall | 149 |  |
| Anal/Frontal Sexual Partners (past 3 months) |  |  |
| 0–1 partners | 42 | 28.2 |
| 2–9 partners | 73 | 50 |
| >10 partners | 34 | 22.8 |
| Oral Sexual Partners (past 3 months) |  |  |
| 0–1 partners | 24 | 16.1 |
| 2–9 partners | 82 | 55 |
| >10 partners | 43 | 28.9 |
| Anal/Frontal Sexual positions (past 3 months) |  |  |
| Receptive-only | 39 | 26.2 |
| Insertive-only | 34 | 22.8 |
| Both insertive/receptive | 69 | 46.3 |
| Oral-only | 7 | 4.7 |
| Condom use with casual partners (past 3 months) |  |  |
| Always | 9 | 6.0 |
| Very often | 10 | 6.7 |
| Sometimes | 27 | 18.1 |
| Rarely | 44 | 29.5 |
| Never | 59 | 39.6 |
| Group sex (past 12 months) |  |  |
| Never | 53 | 35.6 |
| Once | 30 | 20.1 |
| A few times | 55 | 36.9 |
| About once a month or more | 11 | 7.4 |
| Chemsex (past 12 months) |  |  |
| Never | 103 | 69.1 |
| Once | 7 | 4.7 |
| A few times | 33 | 22.2 |
| About once a month or more | 6 | 4.0 |
| Sex on premises venue use |  |  |
| Never | 60 | 40.3 |
| Once | 20 | 13.4 |
| A few times | 57 | 38.3 |
| About once a month or more | 12 | 8 |
| I worry about getting an STI |  |  |
| Strongly Agree | 47 | 31.5 |
| Agree | 67 | 45 |
| Neither agree nor disagree | 18 | 12.1 |
| Disagree | 13 | 8.7 |
| Strongly disagree | 4 | 2.7 |
| Getting an STI could seriously affect my health |  |  |
| Strongly Agree | 24 | 16.1 |
| Agree | 58 | 38.9 |
| Neither agree nor disagree | 32 | 21.5 |
| Disagree | 28 | 18.8 |
| Strongly disagree | 7 | 4.4 |
| Getting an STI is no big deal |  |  |
| Strongly Agree | 10 | 6.7 |
| Agree | 39 | 26.2 |
| Neither agree nor disagree | 38 | 25.5 |
| Disagree | 44 | 29.5 |
| Strongly disagree | 18 | 12.1 |
| I feel I am unlikely to get an STI |  |  |
| Strongly Agree | 10 | 6.7 |
| Agree | 15 | 10.1 |
| Neither agree nor disagree | 36 | 24.1 |
| Disagree | 53 | 35.6 |
| Strongly disagree | 35 | 23.5 |
| I can’t picture myself getting an STI |  |  |
| Strongly Agree | 2 | 1.3 |
| Agree | 8 | 5.4 |
| Neither agree nor disagree | 24 | 16.1 |
| Disagree | 57 | 29.3 |
| Strongly disagree | 58 | 38.9 |
| It is important to me to avoid STIs |  |  |
| Strongly Agree | 59 | 39.6 |
| Agree | 72 | 48.3 |
| Neither agree nor disagree | 16 | 10.7 |
| Disagree | 1 | 0.7 |
| Strongly disagree | 1 | 0.7 |
| Getting an STI is something I think about often |  |  |
| Strongly Agree | 21 | 17.5 |
| Agree | 48 | 32.2 |
| Neither agree nor disagree | 35 | 23.5 |
| Disagree | 33 | 22.2 |
| Strongly disagree | 7 | 4.7 |

Table S2: Sexual behaviour and STI attitudes
