# Supplementary material for: Awareness, attitudes and early use of doxycycline prophylaxis among gbMSM in Ireland: findings from a 2025 community-based cross-sectional survey
Source: Sex Transm Infect. 2026 Jan 14;102(6):e056745. doi: 10.1136/sextrans-2025-056745 (PMC13539834; doi:10.1136/sextrans-2025-056745)
Supplement: Supplementary file 4 [file sextrans-102-6-s003.docx]

# Tables Awareness, Attitudes, and Early Use of Doxycycline Prophylaxis among gbMSM in Ireland: Findings from a 2025 community-based cross-sectional Survey

## Table S3 Awareness, attitudes and use of antibiotic STI prophylaxis

| Variable | n | % |
| --- | --- | --- |
| Awareness of antibiotic STI prophylaxis | 149 |  |
| Awareness of antibiotic STI prophylaxis | 124 | 83.2 |
| Source of information on antibiotic STI prophylaxis* | 124 |  |
| Friend | 67 | 54 |
| Social media | 59 | 47.6 |
| Online community group | 44 | 35.5 |
| Sexual health clinician | 35 | 28.2 |
| Community organisation | 35 | 28.2 |
| Sexual partner | 24 | 19.4 |
| News outlet | 21 | 16.9 |
| Other clinician | 7 | 5.7 |
| Other | 6 | 4.8 |
| Interest in antibiotic STI prophylaxis |  |  |
| Extremely interested in using prophylaxis | 103 | 69.1 |
| Moderately interested in antibiotic STI prophylaxis | 21 | 14.1 |
| Slightly interested in antibiotic STI prophylaxis | 19 | 12.8 |
| Not interested at all | 6 | 4 |
| Concerns about antibiotic STI prophylaxis * | 149 |  |
| Costs of antibiotics | 61 | 40.9 |
| Inconvenience | 87 | 58.4 |
| Adherence | 16 | 10.7 |
| Privacy | 13 | 8.7 |
| Short-term side effects | 45 | 30.2 |
| Long-term side effects | 58 | 38.9 |
| Antibiotic resistance | 104 | 69.8 |
| Lack of information | 15 | 10.1 |
| Not having enough risk | 12 | 8.1 |
| Other | 7 | 4.7 |
| Antibiotic STI prophylaxis use | 149 |  |
| Ever used antibiotic prophylaxis | 44 | 29.5 |
| Used antibiotic prophylaxis past 12m | 42 | 28.2 |
| Antibiotics used  *  ** | 44 |  |
| Doxycycline | 43 | 97.7 |
| Azithromycin | 5 | 11.8 |
| Erythromycin | 1 | 2.3 |
| Amoxicillin | 3 | 6.8 |
| Ceftriaxone | 2 | 4.5 |
| Penicillin | 2 | 4.5 |
| Sources of Antibiotics  *  ** | 44 |  |
| Sexual Health Clinic | 18 | 40.9 |
| Non-Sexual Health Clinic | 4 | 9.1 |
| Online (prescribed) | 12 | 27.3 |
| Online (not prescribed) | 10 | 22.7 |
| Leftover antibiotics from STI | 1 | 2.3 |
| Leftover antibiotics from non-STI | 4 | 9.1 |
| Friend/partner | 11 | 25 |
| Other | 7 | 15.9 |
| STIs aiming to avoid with antibiotic STI prophylaxis  *  ** | 44 |  |
| Chlamydia | 32 | 72.7 |
| Gonorrhea | 31 | 70.5 |
| Syphilis | 27 | 61.4 |
| All STIs | 22 | 59 |
| Other | 1 | 2.3 |
| HIV PrEP use with STI prophylaxis ** | 44 |  |
| STI prophylaxis use on HIV PrEP | 38 | 86.4 |
| Condom use change (while on antibiotic STI prophylaxis ) ** | 44 |  |
| Condom use decreased | 5 | 11.4 |
| Condom use increased | 3 | 6.8 |
| Condom use unchanged | 36 | 81.8 |
| Dosing of DoxyPEP *** | 42 |  |
| 200mgs (recommended) | 41 | 97.6 |
| Unsure | 1 | 2.4 |

Table S3 Awareness, attitudes and use of antibiotic STI prophylaxis

*Participants may select more than one option

**Only those who reported antibiotic STI prophylaxis

*** Only those who reported DoxyPEP use
